# Blockage of Orai1-Nucleolin interaction meditated calcium influx attenuates breast cancer cells growth

**DOI:** 10.1038/s41389-022-00429-z

**Published:** 2022-09-15

**Authors:** Chunming Gu, Wenhao Zhang, Enze Yang, Congyou Gu, Zhaoyang Zhang, Jing Ke, Xiong Wang, Shengying Wu, Shan Li, Fuyun Wu

**Affiliations:** 1 School of Basic Medical Sciences, Hubei University of Medicine, Shiyan, Hubei 442000 China; 2 Hubei Key Laboratory of Embryonic Stem Cell Research, Hubei University of Medicine, Shiyan, Hubei 442000 China; 3 Department of Pathology, The First Affiliated Hospital of Bengbu Medical College, Bengbu, Anhui 100191 China

**Keywords:** Cell growth, Breast cancer

## Abstract

As an important second messenger, calcium (Ca^2+^) regulates a wide variety of physiological processes. Disturbance of intracellular calcium homeostasis implicated in the occurrence of multiple types of diseases. Orai1 is the major player in mediating store-operated calcium entry (SOCE) and regulates calcium homeostasis in non-excitable cells. Over-expression and activation of Orai1 have been reported in breast cancer. However, its molecular mechanisms are still not very clear. Here, we demonstrated that Nucleolin (NCL) was a novel interacting partner of Orai1. NCL is a multifunctional nucleocytoplasmic protein and is upregulated in human breast tumors. The binding of C-termini of NCL (NCL-CT) to N-termini of Orai1 (Orai1-NT) is critical for mediating calcium influx and proliferation of breast cancer cells. Blocking the NCL-Orai1 interaction by synthesized Orai1 peptide can effectively reduce the intracellular calcium influx and suppress the proliferation of breast cancer cells in vitro and in vivo. Our findings reveal a novel activation mechanism of Orai1 via direct interaction with NCL, which may lead to calcium homeostasis imbalance and promote the proliferation of breast cancer cells. Blocking NCL-Orai1 interaction might be an effective treatment of breast cancer.

## Introduction

Breast cancer is one of the most frequent malignancies in women and seriously threatens women’s health [1]. The pathogenesis of breast cancer is quite complicated. Accumulating evidence has demonstrated that calcium homeostasis imbalance and abnormal calcium signal are closely associated with the development of breast cancer [2–4]. Calcium, acting as the crucial second messenger, regulates multiple biological processes, including cell proliferation, migration, apoptosis, and gene transcription [5, 6]. So, intracellular calcium concentration is strictly regulated by membrane calcium channels, calcium pumps, and calcium-binding proteins.

SOCE is one of the main pathways of extracellular calcium influx in mammals, as well as the central mechanism of calcium signal regulation in non-excitable cells [7, 8]. It has been reported that augmented SOCE plays pivotal roles in regulating proliferation, invasion and metastasis of cancer cell [9–11]. SOCE is activated by depletion of intracellular Ca^2+^ stores and mediated by the interaction between the endoplasmic reticulum (ER)-located stromal interaction molecule 1 (STIM1) protein and the plasma membrane (PM)-embedded Orai1 at ER-PM junctions [12–14]. Dysfunction of STIM1/Orai1-mediated SOCE has been found in various tumor types, including breast cancer [15–20]. Orai1, a key component of SOCE, is composed of four transmembrane domains with short intracellular N- and C-terminal, which are involved in interaction with STIM1. However, besides STIM1, Orai1 has been reported to interact with different proteins implicated in tumorigenesis directly [21]. The Secretory Pathway Ca^2+^ ATPases-2 (SPCA2) interacted with Orai1 and activated store-independent calcium influx, promoting breast cancer development and progression [22]. Calcium release activated channel regulator 2A (CRACR2A) regulates the activation of SOCE by interacting with Orai1 and STIM1 directly [23]. STIM1-modulator store-operated calcium entry associated regulatory factor (SARAF) has been demonstrated to activate Orai1 by its interaction with the C-terminus of Orai1 [24]. These findings indicate that Orai1 may be activated by multiple mechanisms.

NCL is a nucleolar phosphoprotein, which mainly distributes in the nucleolus, but also localizes in the nucleoplasm, cytoplasm and cell surface. NCL plays pivotal roles in ribosome biogenesis, chromatin organization and stability, rDNA transcription and RNA metabolism [25, 26]. NCL has been shown to be associated with the development of cancer, and overexpression of NCL is observed in a variety of malignant cancers, which may contribute to tumorigenesis by increasing ribosome assembly to maintain high levels of protein synthesis [27–29]. NCL can also promote the transcription of oncogenes and the activation of signals related to tumor proliferation [30]. NCL also plays an essential role in anti-apoptotic by binding to the mRNA of anti- apoptotic genes and mediating their stability or translation [31]. Mammalian NCL is composed of 710 amino acids containing three domains: a highly acidic N-terminal domain, arginine/glycine-rich (GAR) C-terminal domain, and central domain containing four RNA binding motifs [32]. NCL has been reported to be a calcium-binding protein, and the highly acidic N-terminal may contribute to binding Ca^2+^ [33]. However, the Ca^2+^-related functions of NCL are still unknown. Cell surface NCL was involved in regulating calcium entry into mammalian cells and calcium-dependent ligand internalization [34, 35]. Whether NCL participates in tumor progression by regulating calcium-related actions has not been reported.

In this study, we found that NCL could interact directly with Orai1 inducing calcium influx and promoting breast cancer cells proliferation. Blockage of Orai1-NCL interaction attenuates breast cancer cells growth in vitro and in vivo. Our findings reveal a new activation mechanism of Orai1 and new functions of NCL, which provide a new strategy in breast cancer treatment.

## Materials and methods

### Plasmids and peptides

The pcDNA4/TO-NCL (4TO-NCL) and GFP-Orai1 plasmids were described previously [36, 37]. NCL (residues 284–710, residues 630–710) were cloned into the pGEX-6P-1 vector to generate GST-NCL fragments. Flag-NCL fragments (residues 1–283, residues 284–629, residues 284–710, residues 630–710) were PCR amplified from 4TO-NCL and cloned into the pcDNA4/TO vector. GFP-Orai1 R91W mutant plasmid was created by using the overlap extension PCR site-directed mutagenesis technique. Biotin-labeled Orai1 peptides and cell-penetrating (trans-activator of transcription) TAT-Orai1 peptides were purchased from GL Biochem (Shanghai, China).

### Cell culture and transfection

Human breast cancer MCF-7 and MDA-MB-231 cell lines were obtained from the National Collection of Authenticated Cell Cultures. All the cell lines were authenticated by short tandem repeat (STR) profiling and tested for mycoplasma contamination. MCF-7 cells were cultured in Dulbecco’s modified Eagle medium (DMEM), and MDA-MB-231 cells were cultured in L15 medium supplemented with 10% fetal bovine serum (Hyclone). Cells were transfected using Lipo8000 (Beyotime, China). For stable expression of NCL in breast cancer cells, the NCL gene was cloned into the lenti-CMV vector to package lentiviruses. Breast cancer cells were infected with lentiviruses expression control or NCL-Flag and treated with puromycin. The selected cells were used to assay proliferation and migration of cancer cells.

### Protein expression

E. coli strain BL21 cells containing pGEX-6P-1-NCL were cultured at 37 °C in LB medium to an OD 600 of 0.8 then induced with 0.3 mM isopropyl β-D-thiogalactoside at 25 °C for 16 h. Cells were harvested and lysed by sonication. The GST-NCL proteins were purified by Glutathione Sepharose 4FF (GE healthcare).

### Biotin-based pull-down assay

Biotinylated Orai1-NT or CT peptides were immobilized on streptavidin beads, then mixed with the MCF-7 cell lysate expression NCL-Flag fragments or purified GST-NCL proteins. Beads were washed three times in PBS containing 0.1% Triton X- 100. Proteins were eluted from the beads with 2xSDS sample buffer, then resolved by SDS-PAGE.

### Western blotting

The whole cell lysates were extracted using RIPA buffer, and the protein concentration was determined using the BCA protein assay. Samples were resolved by SDS-PAGE and analyzed by standard Western blotting. The following antibodies were used and purchased from Proteintech Group. Rabbit polyclonal antibodies against NCL (10556-1-AP), Flag (80010-1-RR), PCNA (10205-2-AP), STIM1 (11565-1-AP), PKC (21991-1-AP), AKT (10176-2-AP); Mouse monoclonal antibody against Orai1 (66223-1-Ig), GAPDH (60004-1-Ig). Antibody against Phospho-AKT (CST, 4060) and Phospho-PKC (CST, 9375) were purchased from Cell Signaling Technology.

### Co-immunoprecipitation

The Co-IP assay was performed in MCF-7 cells transfected with NCL-Flag vector using protein A/G magnetic beads (Thermo scientific). After cells were lysed in IP buffer, Orai1 was immuno-precipitated from cell lysate using Flag antibody, and mouse IgG antibodies were used as a negative control. Bound proteins and cell lysate were resolved by SDS-PAGE, followed by western blotting using anti-Flag and anti-Orai1 antibodies.

### Immunofluorescence

MCF-7 cells were plated on confocal glass-bottomed dishes and co-transfected with GFP-Orai1 and NCL-Flag fragments plasmids with or without thapsigargin (TG) treatment. Cells were fixed with 4% paraformaldehyde for 15 min, permeabilized with 0.1% Triton X-100 for 10 min, and blocked with 3% BSA. Cells were stained with Alexa-Fluor-594-conjugated Flag antibody (Proteintech, CL594–66008). Nuclei were counterstained with DAPI for 10 min. Images were taken from confocal microscope.

### Immunohistochemistry

Breast cancer patient tissues were from the first affiliated hospital of Bengbu medical college. The tissues were dehydrated and embedded in paraffin, and then tissues blocks were sectioned at 4-μm thickness for hematoxylin and eosin (H&E) or immunohistochemistry (IHC) analysis. For IHC analysis, tissues were stained with anti-NCL antibody (Proteintech, China) according to the manufacturer’s instruction. Images were randomly taken at a magnification of 400x using a light microscope and analyzed using the ImageJ software.

### siRNA transfection

MCF-7 cells were seeded in a 6-well plate and transfected with 5 μg siRNA with Lipo 8000 reagent. The following siRNA were used: negative control siRNA (siNC) (5′-UUCUCCGAACGUGUCACGU-3′), small interfering RNA for NCL (siNCL) (5′-UUUCUCAAACGAAGUAAGCUUdTdT-3′) and siRNA for STIM1(siSTIM1) (5′-GGCUCUGGAUACAGUGCUCdTdT-3′). Gene silencing was detected by western blotting.

### Determination of changes in intracellular calcium

Calcium imaging was performed as described previously [37]. After transfected, HEK293T cells were loaded with 2 μM Fura-2 AM for 30 min. Cells were excited at 340 nm and 380 nm, and F340/F380 ratios were calculated. For measurement of intracellular calcium using confocal microscopy, cells were plated on confocal glass-bottomed dishes. Cells were then loaded with CalbryteTM 630 AM (final concentration 10 μM) for 40 min. Cells were washed three times in calcium-free buffer. Calcium level (red fluorescence, Ex/Em = 610/640 nm) and GFP signal (green fluorescence, Ex/Em = 488/510 nm) were analyzed with a confocal microscope. The initial fluorescence was recorded for 30 s, then 2 mM CaCl_2_ and 1 μM TG were added, and cellular fluorescence intensity was recorded every 10 s for 5 min.

### Cell proliferation assay

MTT (3- [4,5-dimethylthiazol-2-yl]-2,5 diphenyl tetrazolium bromide) and EdU (5-ethynyl-2′-deoxyuridine) assay were done on MCF-7 or MDA-MB-231cells with stable expression of NCL as described previously [36]. Cells were plated into 96-well plate or 24-well plate for 24 h, then treated with 2-Aminoethoxydiphenyl borate (2-APB) or peptide for 24 h. The cell viability was assessed using MTT assay kit and Edu staining proliferation kit (KeyGEN, China) based on the manufacturer’s instructions.

### Colony formation assay

200 MCF-7 cells with stable expression of NCL were plated into 12-well plates. After being cultured for 7 days, cell clones were treated with 2-APB for 7days. Then all clones were fixed with 4% paraformaldehyde, followed by staining with crystal violet for 5 min. Finally, clones were washed with PBS and photographed.

### Wound healing assay

The transfected cells were plated into a 6-well plate and allowed to grow to a monolayer. The monolayer was then scratched and washed with PBS to remove the detached cells. Cells were maintained in media with or without 2-APB (50 μM) for 24 h. The wounded areas were imaged, and the change in the scratch gap was measured.

### Xenograft animal model

BALB/c nude mice (female, 6 weeks old) were purchased from Wei-tong Lihua experimental animal technical company (Beijing, China). The mice were housed in a specific pathogen-free environment. To establish a breast cancer xenograft model, MCF-7 breast cancer cells (2 × 10^6^) with stable expression of NC or NCL were injected into the nude mice. When tumor volumes reached about 100 mm^3^, NCL group mice were randomly divided into the untreated group, the 2-APB-treated group (20 mg/kg, three times weekly, ip.) and the Orai1 peptide-treated group (50 mg/kg, once daily, ip.), (*n* = 5 mice per group). Mice were monitored up to 14 days after initiation of treatment. Tumor size was measured using digital calipers, and volumes were calculated according to the formula: V (mm^3^) = (width^2^ × length)/2. Tumor weights were measured using an electronic scale after the mice were sacrificed.

### Statistical analysis

Statistical significance was evaluated with data from at least three independent experiments. Statistical analysis was performed using the unpaired *t* test or one-way ANOVA (SPSS 20.0, Chicago). Data are presented as the mean ± SD. For all statistical tests, *P* < 0.05 was considered statistically significant.

## Results

### Identification of NCL as a novel interaction partner of Orai1

**Fig. 1:**
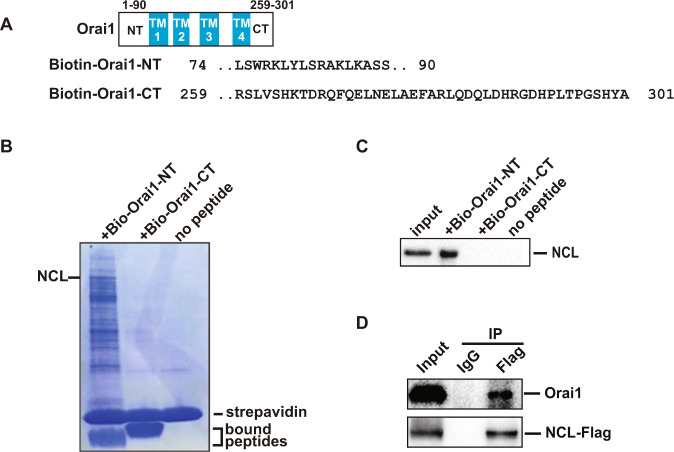
Identification of NCL as a novel interaction partner of Orai1. **A** Domain structures of Orai1 and the synthetic polypeptide sequences of Orai1 terminal. **B** Biotinylated Orai1 peptides were immobilized onto streptavidin beads then mixed with MCF-7 cell lysate to capture the interaction proteins. The samples were analyzed by SDS-PAGE and followed by mass spectrometry. **C** As in B, the pull-down proteins were analyzed by western blotting using anti-NCL antibody. **D** Co-IP assay was performed using MCF-7 cell lysate transfected with NCL-Flag plasmid to test the interaction between Orai1 and NCL. Orai1 was immunoprecipitated from the cell lysate using Flag antibody, and the immunoprecipitated materials were detected by western blotting using anti-Orai1 and anti-Flag antibody. Mouse IgG was used as a negative control in the immunoprecipitation reaction.

The activation and function regulation of Orai1 were meditated through interaction between its N-terminal and C-terminal with many proteins. In order to further investigate potential activation mechanisms of Orai1 involved in the development of breast cancer, biotinylated Orai1-NT or CT peptides were synthesized, and a biotin-based pull-down assay was performed to identify the proteins interacting with Orai1. The length of the peptides was based on previous work [37] (Fig. 1A). Biotinylated Orai1 peptides were immobilized onto streptavidin beads then mixed with MCF-7 cell lysate. The captured proteins were analyzed by SDS-PAGE, and followed by mass spectrometry (Fig. 1B). NCL was identified as a specific interacting protein of Orai1-NT and western blotting analysis also showed that Orai1-NT co-precipitated with the NCL (Fig. 1C). To further confirmed the interaction, a Co-IP assay was performed using anti-Flag antibody after transfection of MCF-7 cells with Flag-tagged NCL. Western blotting analysis revealed the binding of Flag-NCL and endogenous Orai1 (Fig. 1D). Altogether, these results suggested that NCL was a novel binding partner of Orai1, and the interaction may contribute to the progression of breast cancer.

### Upregulation of NCL is associated with the progression of breast cancer

**Fig. 2:**
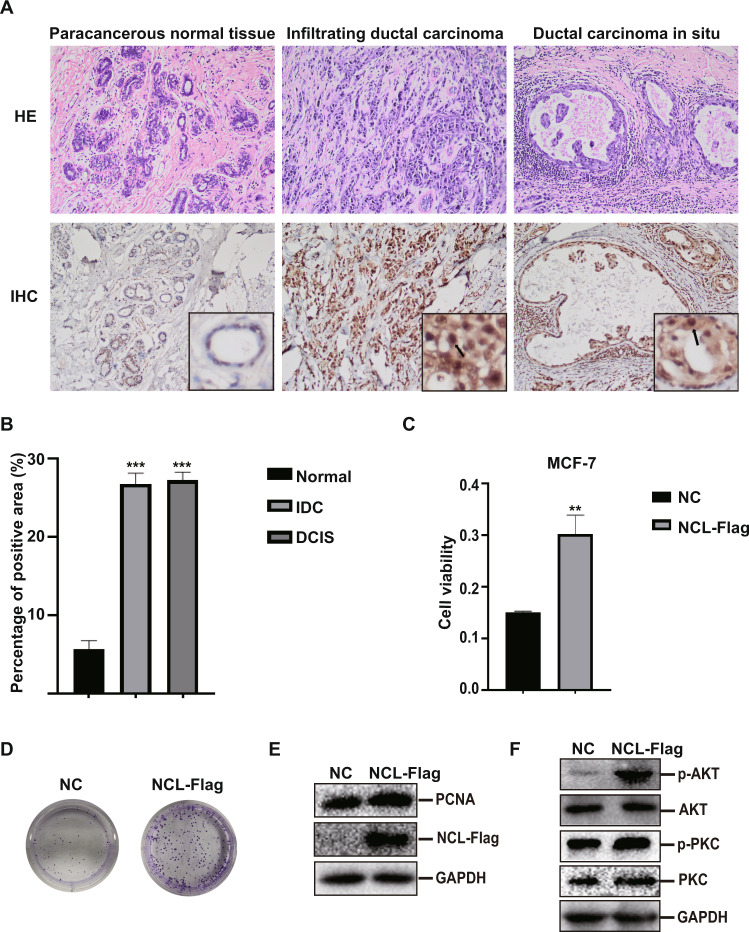
NCL expression is associated with breast cancer progression. **A** H&E staining of breast cancer tissue and immunohistochemical staining using rabbit anti-human NCL antibody, and representative images of NCL expression in normal breast, IDC and DCIS. Magnification is 400X. The enlarged image is shown in the lower right corner. **B** Quantitative analysis of NCL expression in breast tissue. The immunostaining of NCL was quantified based on tissue staining intensity and positive percentage. ***P < 0.001. **C** MTT assay assessed the viability of MCF-7 cells with stable expression of NCL. **P < 0.01. **D** Colony formation assay determined the colony forming ability of MCF-7 cells with stable expression of NCL. **E** Western blotting analysis the expression of PCNA in NCL overexpression MCF-7 cells. **F** As in **E**, Western blotting analysis the expression of AKT, p-AKT, PKC and p-PKC proteins.

To determine the relevance of NCL in breast cancer, we analyzed NCL expression in normal breast tissue, infiltrating ductal carcinoma (IDC) and ductal carcinoma in situ (DCIS) by immunohistochemistry. We found that NCL protein was localized in both nuclei and cytoplasm. The positive expression of NCL was found in both normal tissue and tumor. To our surprise, the NCL expression was significantly higher in breast cancer tissues when compared to the adjacent nonmalignant tissue (Fig. 2A, B). To better understand the function of NCL, we over-expressed NCL in breast cancer cells and examined the effect on cell proliferation. MCF-7 cells transfected with NCL-overexpression plasmid exhibited a significantly higher proliferation rate than the control as assessed by MTT and colony formation assays (Fig. 2C, D). Similarly, we also observed that overexpression of NCL promoted the proliferation of MDA-MB-231 cells (Fig. S1). Proliferating cell nuclear antigen (PCNA) plays an important role in cancer development and progression. We found that overexpression of NCL promoted the expression of PCNA by western blotting analysis (Fig. 2E). Also, we showed that Ca^2+^-dependent protein kinase C (PKC) and protein kinase B (AKT/PKB) pathways were highly activated in NCL overexpression cells (Fig. 2F). Our results indicated that NCL was involved in the progression of breast cancer, possibly by activating calcium-related signaling pathways.

### Mapping the NCL-Orai1 interaction domains

**Fig. 3:**
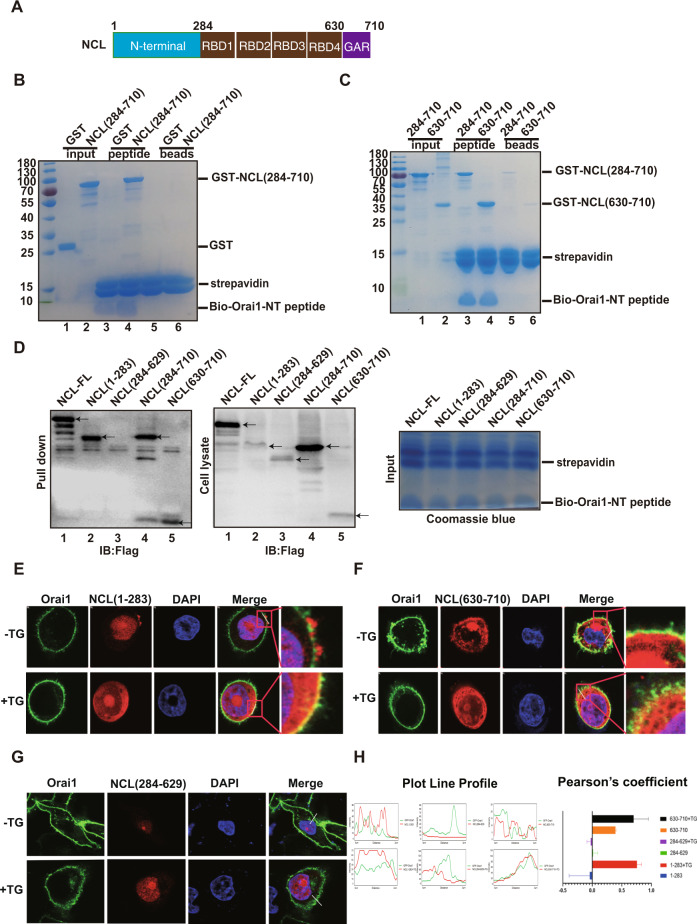
Mapping the NCL-Orai1 interaction domains. **A** Schematic structure of NCL protein. B Biotin-based peptide pulldown assay to determine Orai1-binding domain of NCL. Purified GST and GST-NCL (284–710) proteins were incubated with the immobilized Orai1-NT peptides, then the precipitated samples were analyzed by SDS-PAGE. **C** Biotin-Orai1 peptide pulldown assay with different fragments of GST-NCL. **D** As in **B**, but with the MCF-7 cell lysate transfected with different NCL-Flag plasmids. The precipitated samples and cell lysates were detected by western blotting and bound peptides were analyzed by Coomassie staining. **E** Co-localization of GFP-Orai1 (green) with NCL (1–283)-Flag (red). MCF-7 cells co-transfected with GFP-Orai1 and NCL (1–283)-Flag plasmids were treated with or without TG. The cellular co-localization of Orai1 and NCL was detected by confocal microscopy. The boxed area was shown at high magnification in the panels on the right. **F** As in **E**, but co-localization of GFP-Orai1 with NCL (630–710)-Flag was assayed. **G** As in **E**, but co-localization of GFP-Orai1 with NCL (284–629)-Flag was assayed. **H** Quantification of colocalization between the fluorescent channels of NCL-Flag fragments and GFP-Orai1.

To further investigate whether NCL interacts with Orai1 directly, we performed a in vitro pull-down assay. NCL protein is composed of three domains: N-terminal domain, central RNA binding domain and C-terminal GAR domain (Fig. 3A). Because of containing multiple proteolytic cleavage sites in the N-terminal, full-length NCL is difficult to purify. Therefore, the NT truncated GST-NCL (284–710) fragment and GST proteins were expressed and purified from Escherichia coli, then incubated with Orai1-NT peptides immobilized to the streptavidin-conjugated beads. The bound proteins were analyzed by SDS-PAGE and coomassie staining, which showed an obvious GST-NCL protein precipitated by Orai1-NT (Fig. 3B, lane 4), whereas no precipitation of GST was observed (lane 3) and no binding was observed for beads alone (lane 5,6). Next, we mapped the domain of NCL responsible for interaction with Orai1-NT, whereas the central domain (284–629) of NCL was also difficult to purify. We observed that the purified NCL-CT fragment (630–710) was pulled down by the Orai1-NT peptide (Fig. 3C, lane 4). To further confirm the interaction domain, Flag-tagged full- length NCL and NCL fragments were expressed in MCF-7 cells. Cell lysate was incubated with biotin-Orai1-NT peptide followed by pulling-down with streptavidin beads and western blotting with anti-Flag antibody. Consistently, full-length NCL, NCL-ΔNT (284–710) and NCL-CT (630–710) exhibited interaction with Orai1 (Fig. 3D, lane 1,4, 5, left panel). Unexpectedly, NCL-NT (1–283) binding with Orai1 was also observed (Fig. 3D, lane 2, left panel). The whole cell lysates and bound peptides on the beads were revealed by western blotting and Coomassie staining (Fig. 3D, middle and right panels). Next, we tested the co-localization of NCL fragments with Orai1. MCF-7 cells were co-transfected with GFP-Orai1 and Flag-NCL fragment plasmids, then treated with or without TG, a blocker of sarcoendoplasmic reticulum calcium ATPase (SERCA) pump to induce SOCE, followed by immunofluorescence staining with anti-Flag antibody (red fluorescence). Expression of GFP-Orai1 exhibited its localization in the plasma membrane and NCL (1–283) mainly localized in the nucleoplasm. No obvious Orai1/NCL (1–283) co-localization was observed in the plasma membrane, whereas treatment with TG caused the accumulation of NCL (1–283) in the cytoplasm and plasma membrane which strongly co-localized with Orai1 (Fig. 3E). In addition, NCL (630–710) showed a clear shift in localization towards the cytoplasm and plasma membrane and exhibited strong co-localization with Orai1 in the plasma membrane with or without TG treatment (Fig. 3F). NCL (284–629) was found to localize in the nucleus, where no obvious Orai1/NCL co-localization was observed and no difference in co-localization was observed when cells were treated by TG (Fig. 3G). Furthermore, colocalization in the PM between NCL fragments and Orai1 was quantified (Fig. 3H). Thus, these results are consistent with the pull-down assay and suggest that both the NT and CT of NCL contributed to the interaction with Orai1.

### NCL-Orai1 interaction meditated store-operated calcium entry

**Fig. 4:**
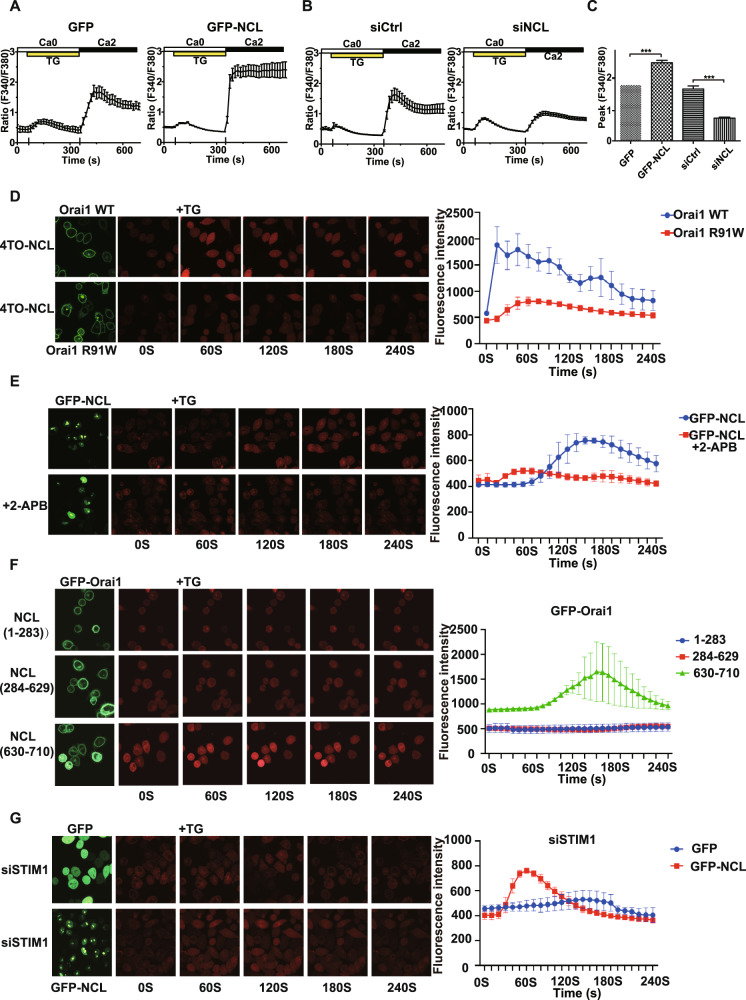
NCL-Orai1 interaction meditated store-operated calcium entry. **A** Representative traces of fura-2 Ca^2+^-imaging experiments in HEK293T cells transfected with GFP or GFP-NCL. Calcium stores were depleted with 1 μM TG, and SOCE was measured by the addition of 2 mM Ca^2+^ in the medium. **B** As in **A**, but HEK293T cells transfected with control siRNA or NCL siRNA. **C** Representative Fura-2 fluorescent ratio in HEK293T cells expressing GFP vector, GFP-NCL, siCtrl and siNCL. ***P < 0.001. **D** Live-cell imaging of intracellular calcium flux using confocal microscopy. MCF-7 cells co-transfected with 4TO-NCL and GFP-Orai1 WT or GFP-Orai1 R91W plasmids were loaded with red-fluorescent calcium indicator CalbryteTM 630 AM and imaged via laser scanning fluorescence confocal microscopy. After treatment with TG, the real-time alterations in intracellular Ca^2+^ concentration was measured. The calcium level in MCF-7 cells was expressed as the relative fluorescence intensity based on the fluorescence images. **E** As in **D**, but cells transfected with GFP-NCL were treated with or without 2-APB, then intracellular calcium flux was measured by confocal microscopy. **F** As in **D**, but cells were co-transfected with GFP-Orai1 and different fragments of 4TO-NCL plasmids. The real-time alterations in intracellular Ca^2+^ concentration was measured by confocal microscopy. **G** As in **D**, but MCF-7 cells transfected with GFP or GFP-NCL, then were transfected with STIM1 siRNA. After treatment with TG, the real-time alterations in intracellular Ca^2+^ concentration was measured. All data are shown as mean ± SD. of three replicates. *P < 0.05. **P < 0.01. ***P < 0.001.

To study the role of NCL-Orai1 interaction in the progression of breast cancer, we tested whether NCL was involved in SOCE. Firstly, we examined the effect of NCL expression level on TG-evoked SOCE in HEK293T cells by calcium imaging. Ca^2+^ influx was measured by monitoring the cytosolic Ca^2+^ levels with Ca^2+^ indicator Fura-2 AM after store depletion induced by TG. We found that when overexpression of GFP-NCL, SOCE was significantly elevated following addition of 2 mM Ca^2+^ compared with GFP control (Fig. 4A). Conversely, knocking down NCL expression by siRNA resulted in a decrease in SOCE compared with the control siRNA group (Fig. 4B, C). We next tested whether NCL regulated SOCE by interaction with Orai1. We also visualized the real-time alterations in the intracellular Ca^2+^ concentration in breast cancer cells through confocal microscopy in conjunction with red fluorescent Ca^2+^ indicator CalbryteTM 630 AM. Consistently, overexpression of GFP-NCL in MCF-7 cells and MDA-MB-231 cells significantly increased basal Ca^2+^ concentration and TG induced SOCE (Fig. S2). Therefore, our data suggested that NCL was implicated in the regulation of SOCE. We next tested whether NCL regulated SOCE by interaction with Orai1. Firstly, MCF-7 cells were co-transfected with 4TO-NCL and GFP-Orai1 WT or R91W mutant plasmids, then TG-evoked SOCE was measured. We observed that when NCL and Orai1 WT were co-expressed, SOCE was obviously elevated, but not when NCL and channel dead mutant R91W were co-expressed (Fig. 4D). Then we tested whether Orai1 channel inhibitor 2-APB affected NCL-meditated SOCE. As expected, the SOCE of GFP-NCL over-expressed cells was reduced after treatment with 2-APB (Fig. 4E). Additionally, we observed a dramatic decrease of TG-evoked SOCE when we depleted NCL using siRNA in cells overexpressing GFP-Orai1 (Fig. S3). Thus, our findings indicated that NCL played a crucial role in the regulation of SOCE via Orai1. To further elucidate the activation mechanism of Orai1 by NCL, the effects of NCL fragment overexpression on SOCE were investigated. We noticed that the N-terminus of NCL did not induce the calcium entry despite being able to interact directly with Orai1, and the central region of NCL did not enhance the SOCE as expected, whereas C-terminus was sufficient to increase basal Ca^2+^ concentration and induce SOCE (Fig. 4F). Next, we tested whether STIM1 is required for NCL-meditated activation of Orai1. STIM1 was knocked down by siRNA in MCF-7 cells (Fig. S4). Depletion of STIM1 did not result in a marked decrease of NCL-meditated SOCE suggesting that activation of Orai1 by NCL was independent of STIM1 (Fig. 4G). Taken together, our results suggested that the C-terminus of NCL interacts with Orai1 to activate Ca^2+^ influx, which may alter calcium-regulated signal and contribute to breast cancer progression.

### NCL activates Orai1 to promote proliferation of breast cancer cells

**Fig. 5:**
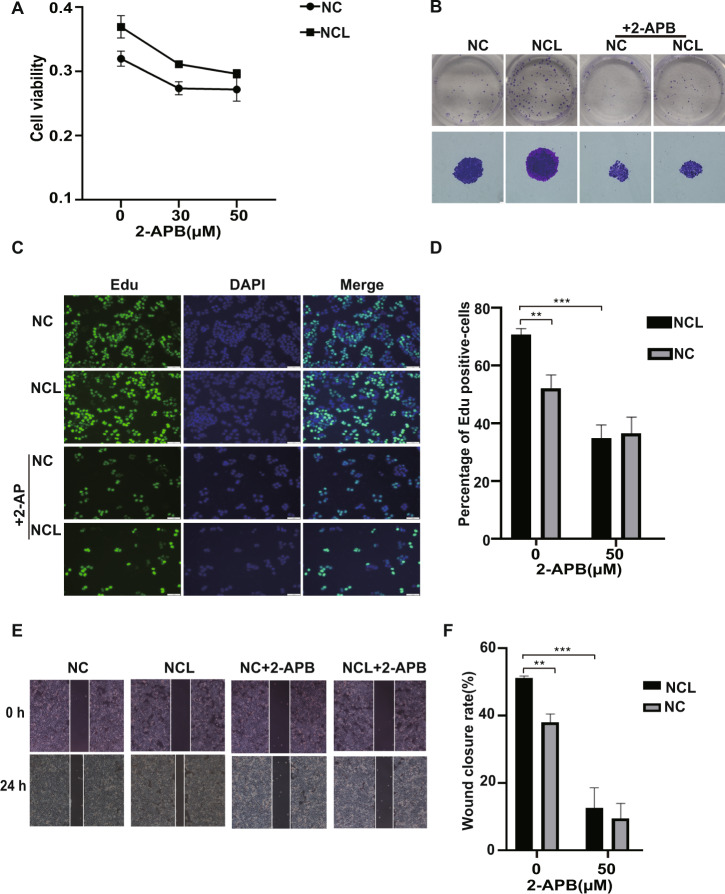
Inhibition of the calcium influx abolished the promoting effects of NCL on the proliferation of breast cancer cells. **A** MTT assay assessed the effect of 2-APB on the proliferation of MCF-7 cells stable expression of NCL. MCF-7 cells stable expression of NCL and negative control cells were treated with different concentration of 2-APB, and then the viability of cells was evaluated. **B** Colony formation assay of MCF-7 cells stable expression of NCL and negative control cells treated with or without 2-APB. **C** EdU staining for evaluation of the influences of 2-APB on the proliferation of NCL-overexpression MCF-7 cells. Dividing cells were labeled with EdU (green). All cells were counterstained with DAPI (blue). **D** Quantitative analyses of the percentages of EdU-positive cells. **E** Scratch assay to evaluate migration ability of MCF-7 cells stable expression of NCL and negative control cells. After cell growth reached confluence, a scratch was made by a 200 μL pipette tip, and cells were treated with or without 2-APB for 24 h. The closure of the scratch was imaged and analyzed. **F** Wound closure rates were expressed as percentages of the wound area closed at 24 h relative to the initial area at 0 h. All data are shown as mean ± SD. of three replicates. *P < 0.05. **P < 0.01. ***P < 0.001.

To probe the linkage between NCL-meditated SOCE and the proliferation of breast cancer cells, we established the stable breast cancer cell lines (MCF-7 and MDA-MB-231) with constitutive overexpression of NCL and performed MTT assay (Fig. 5A), colony formation (Fig. 5B) and EdU proliferation assays (Fig. 5C, D). These assays revealed that NCL overexpression promoted proliferation and growth of breast cancer cells, but treatment with Orai1 channel blocker 2-APB abolished the promoting effects of NCL on cell proliferation and clonogenicity. In addition, wound healing assay showed the promoting effect of NCL on cell migration, but inhibition of SOCE with 2-APB significantly decreased migration ability that was promoted by NCL (Fig. 5E, F). A similar inhibition effect of 2-APB on NCL-meditated proliferation and migration was observed in MDA-MB-231 cells (Fig. S5). Our results suggested that NCL promoted the proliferation of breast cancer cells by increasing calcium influx.

### Blocking the interaction between NCL and Orai1 with synthesized Orai1 peptide inhibited breast cancer cells proliferation

**Fig. 6:**
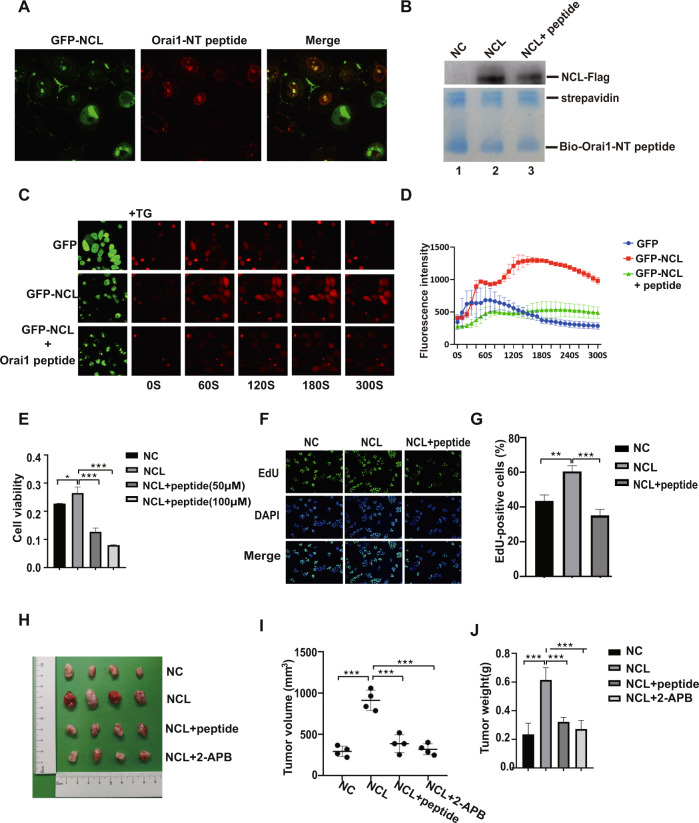
Blocking the interaction between NCL and Orai1 with Orai1 peptide inhibited breast cancer cells proliferation in vitro and in vivo. **A** Co-localization of GFP-NCL (green) with rhodamine-labeled TAT-Orai1-NT peptide (red). MCF-7 cells transfected with GFP-NCL were incubated with Orai1 peptide for 1 h, then the cellular co-localization of TAT-Orai1-NT peptide and NCL was detected with confocal microscopy. **B** Biotin-based peptide pulldown assay to determine peptide-blocking effect of interaction between NCL and Orai1. MCF-7 cells transfected with NCL-Flag plasmid were incubated with the cell-penetrating TAT-Orai1-NT peptides. The cell lysate was bound with biotinylated Orai1-NT peptides immobilized on streptavidin beads and precipitated NCL proteins were detected by western blotting using anti-Flag antibody. **C**, **D** Measurement intracellular calcium influx using confocal microscopy in MCF-7 cells transfected with GFP-NCL and treated with Orai1-NT peptide. **E** MTT assay to assess the effect of Orai1-NT peptide on the proliferation of MCF-7 cells stable expression of NCL. **F**, **G** EdU staining for evaluation of the influences of Orai1 peptide on the proliferation of breast cancer cells stable expression of NCL. **H** Subcutaneous xenograft tumor growth in nude mice was measured. MCF-7 cells stable expression of NCL and negative control cells were transplanted to BALB/c nude mice. Mice were treated with Orai1 peptides or 2-APB. Tumor volume and weight were measured (**I**, **J**). All data are shown as mean ± SD. of three replicates. *P < 0.05. **P < 0.01. ***P < 0.001.

NCL-meditated SOCE was required for the proliferation of breast cancer cells, so blocking the binding between NCL and Orai1-NT may lead to the reduction in calcium influx and inhibition of cancer cells proliferation. To investigate this possibility, the Rhodamine-labeled TAT-Orai1-NT peptides were synthesized and loaded in MCF-7 cells to block the interaction (TAT, a cell-penetrating peptide). After Orai1 peptide incubated with MCF-7 cells expressing GFP-NCL, the co-localization of Orai1 peptide and NCL was observed in the cell membrane and nucleus by confocal microscopy (Fig. 6A), which indicates that Orai1 peptide may play the role of disrupting the interaction between NCL and Orai1 by competitively binding NCL in the different cellular compartments. To test this possibility, we still performed the pull-down assay using biotinylated Orai1-NT peptides and cell lysate of MCF-7 overexpressing NCL-Flag with or without TAT-Orai1-NT peptides treatment. As expected, the cell-penetrating Orai1 peptide effectively reduced the co-precipitation of NCL with the biotinylated peptides (Fig. 6B, lane3). Next, we test whether TAT-Orai1 peptide (No coupling rhodamine) inhibited the NCL-meditated SOCE. MCF-7 cells transfected with GFP-NCL exhibited elevated Ca^2+^ influx after the addition of TG and 2 mM Ca^2+^ than cells transfected with GFP, but when treated with Orai1 peptides, the SOCE of GFP-NCL transfected cells was dramatically reduced (Fig. 6C, D). We also investigated whether Orai1 peptides performed the inhibitory effect on the proliferation of breast cancer cells. MTT assay revealed that Orai1 peptides treatment reversed the increased cell proliferation of breast cancer cells induced by NCL overexpression (Fig. 6E). Similarly, the EdU assay results revealed that the percentage of EdU-labeled cells was declined after Orai1 peptides treatment, suggesting peptides abolished the promoting effects of NCL on cell proliferation (Fig. 6F, G). To confirm the hypothesis that NCL promoted tumor growth by increasing calcium influx via interaction with Orai1, we subcutaneously implanted MCF-7 cells stably expression of NCL and negative control cells into nude mice to establish the subcutaneous breast cancer xenograft. NCL group was treated with 2-APB to inhibit the calcium influx or treated with Orai1 peptides to block the NCL-Orai1 interaction. As expected, NCL overexpression markedly promoted the growth of tumor volume and increased tumor weight than the negative control group. Still, both 2-APB treated group and Orai1 peptides treated group displayed a significantly smaller tumor volume compared to the NCL group (Fig. 6H–J). Taken together, these results suggested that NCL-Orai1 interaction-meditated calcium entry was crucial for breast cancer development, and blocking the NCL-Orai1 pathway may be an effective strategy for the treatment of some breast cancer subtypes.

## Discussion

Calcium signaling is an essential mediator of many processes in breast cancer progression. Calcium channels have been considered as potential drug targets in breast cancer. Orai1 has been reported to control tumorigenesis and tumor progression by virtue of the abnormal expression and activation, resulting in the imbalance of intracellular calcium homeostasis [38, 39]. Orai1 is mainly activated by calcium sensor STIM1 when calcium stores are depleted, whereas several interacting partners of Orai1 have been identified and involved in the regulation of calcium signal, suggesting that the activation mechanism and function of Orai1 still need to be clarified. In this research, we found a novel interaction between Orai1 and NCL (Figs. 1, 3).

NCL, an important nucleolar phosphoprotein, is involved in ribosomal biogenesis, rRNA processing and mRNA stability. NCL is mainly distributed in the nucleolus, but also in the nucleoplasm, cytoplasm and cell surface. Overexpression of NCL has been found in a variety of tumors, and each cell compartment of NCL could exert distinct functions in cancer progression. Our findings uncover a critical function of NCL in the development of breast cancer (Fig. 2). Recently, cell surface NCL as a receptor has attracted great attention as a potential target for cancer therapy [40]. AS1411, a DNA aptamer targeting cell surface NCL, has shown potent anti-proliferative activity in various cancer cells and entered phase II clinical trials in multiple advanced cancers [41, 42]. Moreover, various molecules have been identified as cell surface NCL-ligands involved in tumor development [43]. Our studies provided new insights into the role of cell surface NCL in breast cancer. Both the N-terminal and C-terminal of NCL interact with Orai1, which may facilitate the cell surface accumulation of NCL. Overexpression of NCL-CT exhibits a significant increase of SOCE, but the N-terminal of NCL doesn’t affect the TG-evoked SOCE (Fig. 4). NCL has been reported to be a calcium-binding protein, and the highly acidic N-terminal contributes to binding Ca^2+^. So, the N-terminal of NCL may play a function similar to STIM1, sensing the calcium concentration change in the nucleus and assisting the interaction between NCL-CT and Orai1. We also found that knockdown of NCL expression caused the decrease of calcium in the cell nucleus after TG treatment (Fig. S6). So far, the regulatory mechanism of nuclear calcium is still very unclear. We speculate that NCL may play a role in regulating nuclear calcium. When the ER calcium store is depleted and nuclear calcium is reduced, NCL may sense the change of calcium concentration via its N-terminal, resulting in a conformation change and translocation of NCL from the nucleus to the cytoplasm and cell surface where it interacts with Orai1 to meditate calcium influx replenishing the calcium store and nuclear calcium. However, its underlying molecular mechanisms need to be further clarified.

Accumulating evidence of NCL in cancer development and cancer therapy suggests that NCL is a candidate biomarker and a promising target for cancer treatment. Our studies have demonstrated that NCL plays a crucial role in the proliferation of breast cancer cells by regulating the SOCE. Orai1 inhibitor 2-APB can significantly inhibit NCL-meditated calcium influx and abolish the promoting effects of NCL on breast cancer cells proliferation (Fig. 5). More importantly, the synthesized Orai1 peptide specifically targeting NCL and blocking NCL-Orai1 interaction, attenuates the growth of breast cancer cells through the reduction of NCL-meditated SOCE. Move over, peptides exhibit the strong anti-tumor activity in a xenograft mouse model (Fig. 6). Thus, the NCL-Orai1 interaction may be a valuable target for controlling calcium homeostasis and the development of breast cancer.

## Conclusions

**Fig. 7:**
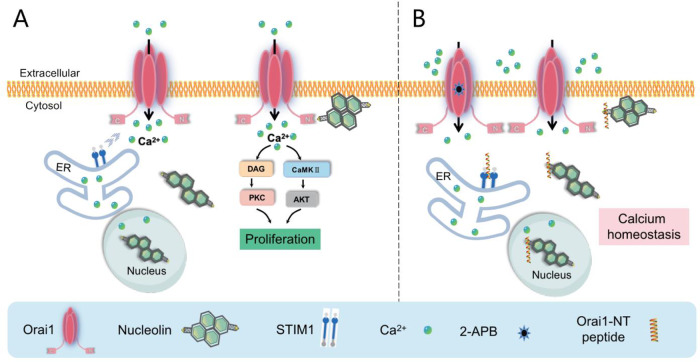
Schematic model for the activation of Orai1 by Nucleolin promotion the proliferation of breast cancer cells. **A** NCL proteins shuttle from nucleolus to cytoplasm and cell membrane, and then interact with calcium channel Orai1 at the plasma membrane via the C-terminal domain, which results in activation of Orai1 channel and calcium entry, eventually leading to activation of calcium related signaling pathways and promotion breast cancer cell proliferation. **B** Orai1 inhibitor 2-APB and synthesized Orai1-NT peptides blocking NCL/Orai1 interaction could inhibit NCL-meditated calcium influx, partially restore intracellular calcium homeostasis and inhibit the proliferation of breast cancer cells.

In summary, we identified NCL as a key regulator for calcium homeostasis in breast cancer cells via activation of Orai1. Blocking the Orai1 channel or NCL-Orai1 interaction inhibits the promoting effects of NCL on breast cancer cells proliferation (Fig. 7). Our results provide the novel potential therapeutic targets for breast cancer treatment.

## Supplementary information

Supplementary material

## Data Availability

The data and materials of this study are available from the corresponding author upon reasonable request.
