# Supplementary material for: Blockage of Orai1-Nucleolin interaction meditated calcium influx attenuates breast cancer cells growth
Source: Oncogenesis. 2022 Sep 15;11(1):55. doi: 10.1038/s41389-022-00429-z (PMC9478099; doi:10.1038/s41389-022-00429-z)
Supplement: Supplementary file 1 — Supplementary material [file 41389_2022_429_MOESM1_ESM.pdf]

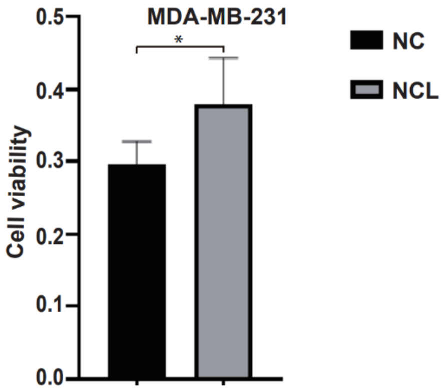

Figure S1. NCL overexpression promoted the proliferation of MDA-MB-231 cells. MTT assay was performed to measure the proliferation of MDA-MB-231 cells with stable expression of NCL. The data is shown as mean ± SD. of three replicates. *, $P < 0.05$.

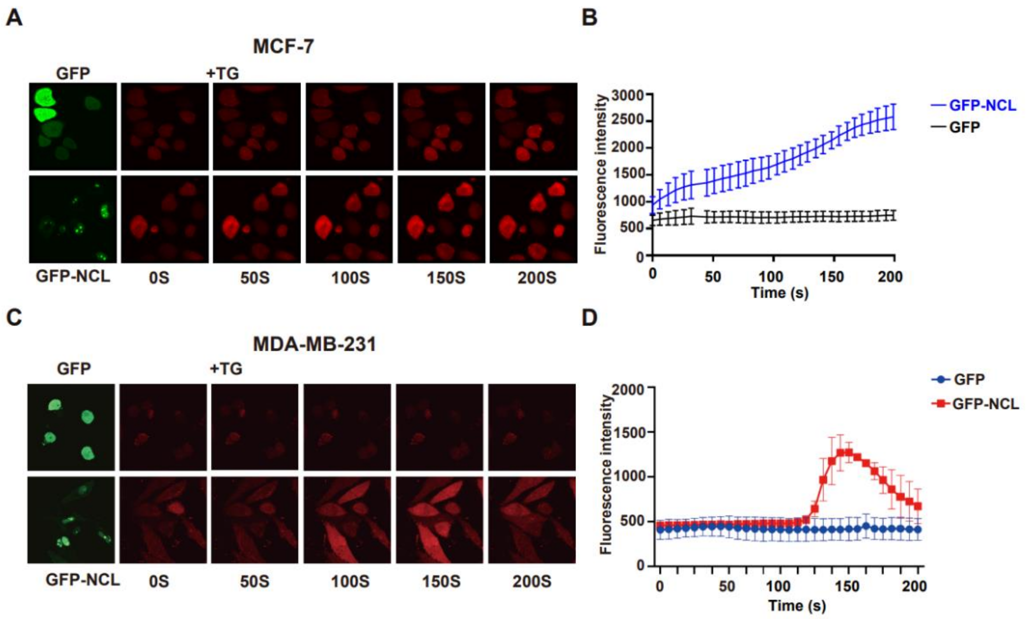

Figure S2. Overexpression of NCL increased TG-evoked SOCE in breast cancer cells. (A, C) Live-cell imaging of intracellular calcium flux using confocal microscopy. MCF-7 cells and MDA-MB-231 cells transfected with GFP or GFP-NCL were loaded with red-fluorescent calcium indicator CalbryteTM 630 AM. After treatment with TG, the real-time alterations in intracellular Ca^{2+} concentration was measured. (B, D) The calcium level in MCF-7 and MDA-MB-231 cells was expressed as the relative fluorescence intensity.

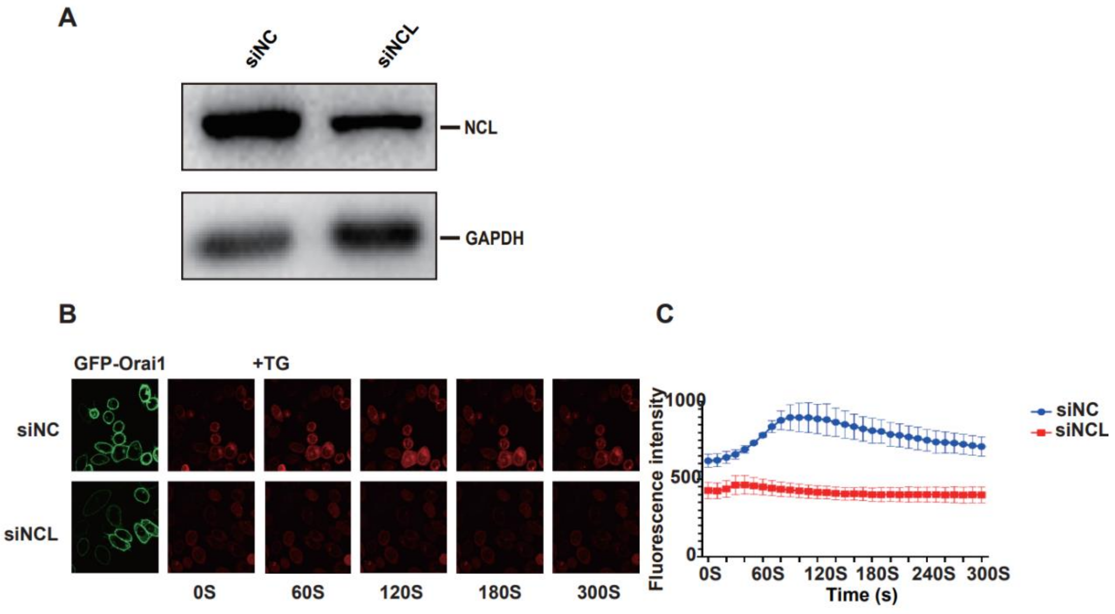

Figure S3. Knocking down NCL expression by siRNA resulted in a decrease in SOCE. (A) Western blot analysis of the efficiencies of siRNA knockdown of NCL expression in MCF-7 cells. GAPDH was used as the loading control. (B) Measurement intracellular calcium flux using confocal microscopy in MCF-7 cells transfected with GFP-Orai1 and depleted NCL using siRNA. (C) The relative fluorescence intensity of calcium indicator in MCF-7 cells based on the fluorescence images in (B).

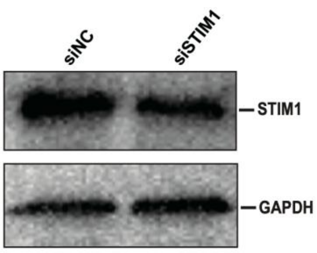

Figure S4. Western blot analysis of the efficiencies of siRNA knockdown of STIM1 expression in MCF-7 cells.

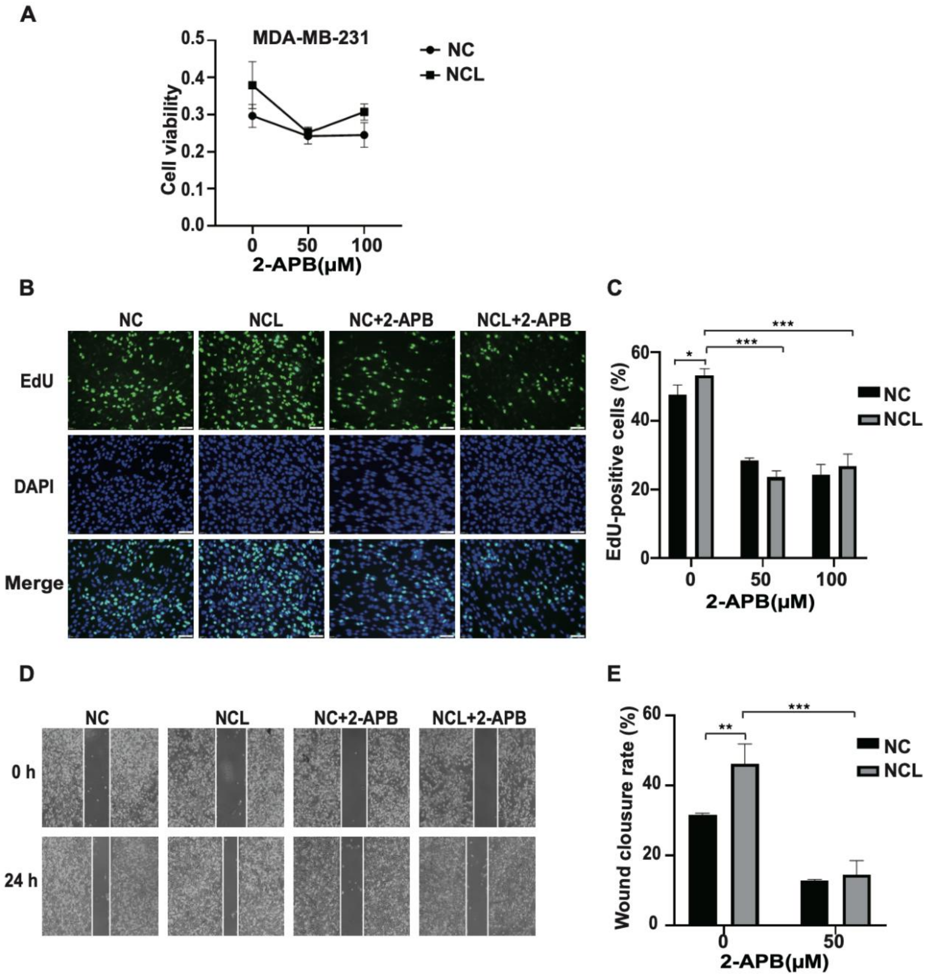

Figure S5. Inhibition of the calcium influx abolished the promoting effects of NCL on the proliferation of MDA-MB-231 cells. (A) MTT assay analysis of the effect of 2-APB on the proliferation of MDA-MB-231 cells stable expression of NCL. (B) EdU staining for evaluation of the influences of 2-APB on the proliferation of MDA-MB-231 cells stable expression of NCL. (C) Quantitative analyses of the percentages of EdU-positive cells. (D) Scratch assay was performed in MDA-MB-231 cells stable expression of NCL and negative control cells. (E) Wound closure rates were expressed as percentages of the wound area closed at 24h relative to the initial area at 0h. All data are shown as mean ± SD. of three replicates. *, $P < 0.05$. **, $P < 0.01$. ***, $P < 0.001$.

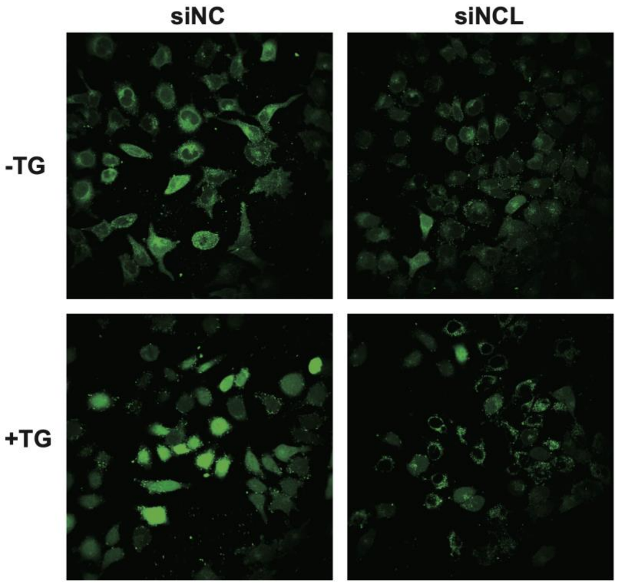

Figure S6. Knocking down NCL expression resulted in a decrease of calcium in the cell nucleus after TG treatment. MCF-7 cells transfected with control siRNA or NCL siRNA were loaded with Fluo4-AM, then the fluorescence intensity of MCF-7 cells was detected with confocal microscopy in the absence or presence of TG.
